# Prospective errors determine motor learning

**DOI:** 10.1038/ncomms6925

**Published:** 2015-01-30

**Authors:** Ken Takiyama, Masaya Hirashima, Daichi Nozaki

**Affiliations:** 1 Brain Science Institute, Tamagawa University, Machida-shi, Tokyo 194-8610, Japan; 2 Center for Information and Neural Networks (CiNet), National Institute of Information and Communications Technology, Osaka University, Suita, Osaka 565-0871, Japan; 3 Graduate School of Education, The University of Tokyo, Bunkyo-ku, Tokyo 113-0033, Japan

## Abstract

Diverse features of motor learning have been reported by numerous studies, but no single theoretical framework concurrently accounts for these features. Here, we propose a model for motor learning to explain these features in a unified way by extending a motor primitive framework. The model assumes that the recruitment pattern of motor primitives is determined by the predicted movement error of an upcoming movement (prospective error). To validate this idea, we perform a behavioural experiment to examine the model’s novel prediction: after experiencing an environment in which the movement error is more easily predictable, subsequent motor learning should become faster. The experimental results support our prediction, suggesting that the prospective error might be encoded in the motor primitives. Furthermore, we demonstrate that this model has a strong explanatory power to reproduce a wide variety of motor-learning-related phenomena that have been separately explained by different computational models.

## Abstract (web summary)

Motor learning is characterized by diverse cognitive processes, which lack a unified theoretical framework. Here, Takiyama *et al.* present a model demonstrating that motor learning is determined by prospective errors, which they test in a specially designed visuomotor adaptation task.

## Graphical abstract

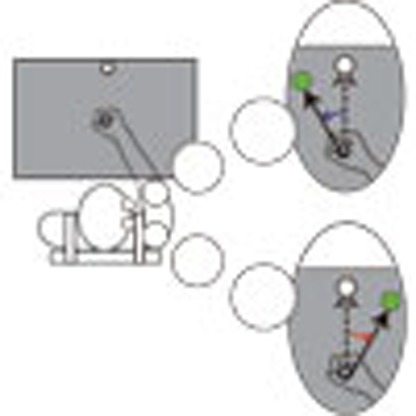

Diverse features of motor learning have been reported by numerous experiments, but no single theoretical framework concurrently accounts for all of these features. For example, after learning in a novel visuomotor environment followed by a washout phase, the learning speed in the relearning phase is faster than that in the initial learning phase. This acceleration of motor learning has been explained by the incorporation of fast and slow components into the motor-learning process^1^. However, it remains unclear how such a multi-learning-rate model can be extended to explain the decrement of learning speed with increased uncertainty of feedback information. Although a standard Kalman filter^2^,^3^,^4^ successfully explains this uncertainty effect, it cannot explain how motor memory can be formed and maintained even when the environment randomly varies from trial to trial (structural learning)^5^,^6^,^7^. Several models have been proposed to explain structural learning by assuming that subjects have already acquired *a priori* knowledge regarding the tendency of environmental variation^8^,^9^. However, to our knowledge, few computational models can explain structural learning without any *a priori* knowledge. Thus, a single framework that can explain such a wide variety of phenomena is currently unavailable.

Here we propose a novel model for motor learning to explain a wide variety of phenomena in a unified way by extending a theoretical framework of motor primitives^10^,^11^,^12^,^13^,^14^,^15^. In the original framework, activities of motor primitives determine motor commands, and an appropriate set of motor primitives is recruited according to the various features of the desired movement, such as planned movement direction^10^,^11^. This framework successfully reproduces the basic pattern of trial-dependent changes in the movement error and how motor learning is generalized when the kinematics (for example, movement direction) change.

However, the manner in which the activities of motor primitives are determined remains controversial. In contrast to the conventional idea that the desired movement direction determines the activities of motor primitives^10^,^11^,^12^, a recent study suggested the possible involvement of the executed movement in determining these activities^13^. The model we propose in the present study assumes that the predicted movement error of an upcoming movement, termed the prospective error (PE), also contributes to determining the activities of the primitives. This assumption is based on two components: (1) a theoretical consideration regarding the formation and maintenance of motor memory from a randomly changing environment, and (2) recent neurophysiological findings^16^,^17^ showing that some motor-related neurons encode the PE rather than the desired or executed movements.

In the present study, first, we analytically reveal that the activities of motor primitives need to be determined based on the PE such that the motor memory can be formed and maintained in a randomly changing environment. Second, to validate the idea of incorporating the PE into motor learning, we experimentally demonstrate a novel motor-learning phenomenon that can be predicted by our model: after experiencing an environment in which the movement error is more easily predictable, subsequent motor learning should become faster. Finally, using a computer simulation, we show that our model can account for several different and seemingly unrelated phenomena in motor learning, such as structural learning^5^,^6^,^7^, modulation of the learning rate because of uncertainty of error feedback^3^,^4^, savings after short and long washout trials^18^,^19^,^20^, anterograde interference^21^,^22^ and spontaneous recovery^1^,^23^,^24^. Although different conventional models have separately explained these phenomena, our model is unique in that it can explain them within a single framework.

## Results

### General framework

**Figure 1:**
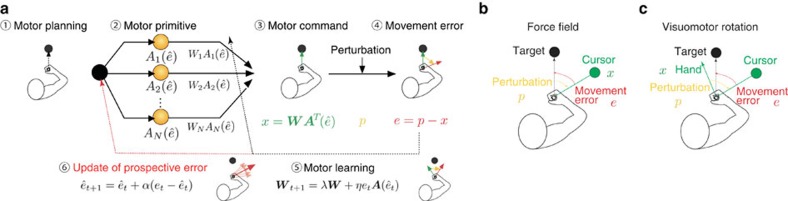
Model schematic. (**a**) A schematic representation of our model. Prospective error determines activities of motor primitives, the weighted sum of these activities determines motor commands and movement error is a learning signal for the weighting parameters and prospective error. (**b**) Force field: subjects need to move the cursor or his/her hand towards the target. (**c**) Visuomotor rotation: subjects need to move the cursor towards the target.

The present study used a task involving reaching towards a single target in a horizontal plane (Fig. 1a). The goal of the task was to move a cursor to the target accurately in a situation where an executed movement is perturbed by a change in the environment, *p*, for example, the external force generated by a manipulandum^25^ (Fig. 1b) or visuomotor transformation^26^ (Fig. 1c). The motor command, *x*, to compensate for a perturbation, *p*, is modelled by the summation of the activities of the motor primitives as *x*=***WA***^*T*^, where ***W***=(*W*_1_, ..., *W*_*N*_), *N* is the total number of motor primitives, *W*_*i*_ represents how the *i*th primitive contributes to the production of the motor command, ***A***=(*A*_1_, ..., *A*_*N*_), and *A*_*i*_ is the activity of the *i*th primitive (we propose that this be determined depending on the PE (details are provided in the section Prospective error)). The movement error at the *t*-th trial can thus be expressed as 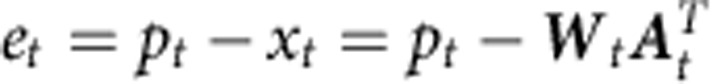. To minimize the squared movement error, ***W*** is modified as


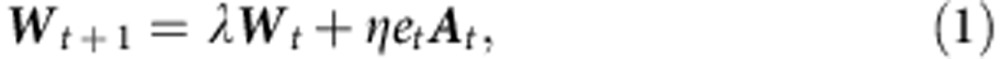


where *λ* is the forgetting rate and *η* is the learning rate, indicating that the more activated the *i*th primitive, the more the *W*_*i*_ is modified to minimize the squared movement error (the stronger the motor memory is formed in the *i*th primitive). Similarly, if the *i*th primitive is not activated at the *t*-th trial, *W*_*i*_ is not modified (the motor memory embedded in the *i*th primitive can be kept).

### Theoretical considerations in randomly changing environments

First, we analytically considered the problem of what characteristics of the movement the primitives need to encode. We focused on the problem of how a motor memory can be formed within a randomly changing environment. Recent works have illustrated the ability of the motor system to form motor memories from randomly changing environments: the experience of a randomly changing visuomotor rotation increased the speed of the subsequent learning to a constant visuomotor rotation (structural learning)^5^,^6^,^7^.

In our model described above, when the perturbation randomly changes from trial to trial, the ensemble average for ***W***_*t*_, ***W***_*t*+1_, ***W***_*t*+2_, ... across all possible realizations converges to


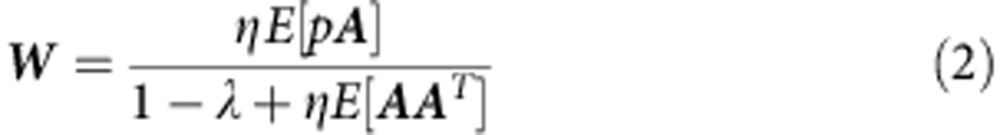


after many trials, where *E*[·] represents the ensemble average taken across different simulation runs (see the Theoretical analysis section in Methods for a detailed analysis). When the perturbation randomly changes around 0, *E*[*p*]=0. If *p* and ***A*** are independent, then the weighting parameter ***W***=0. This indicates that motor primitives can form and maintain motor memory in a randomly varying environment only when ***A***_*t*_ encodes the information of *p*_*t*_.

### Prospective error

Notably, ***A***_*t*_ cannot directly encode *p*_*t*_, because the information for *p*_*t*_ is only available after motor execution. A possible solution is to assume that the motor-learning system predicts a factor (factors) that contains the information of *p*_*t*_. Because the goal of motor learning is to minimize movement error, the motor-learning system uses a movement error, *e*_*t*_, as a learning signal. Here, we assumed that *e*_*t*_ is used not only as a learning signal but also as a signal for predicting the PE (Fig. 1a), which should contain the information regarding the perturbation. Recent neurophysiological studies have suggested that some neurons actually encode the PE, or the movement error predicted to be observed in the near future for online movement control^16^,^17^.

Specifically, we assume that the PE is predicted from both the PE and the observed movement error in the previous (*t*−1)-th trial:


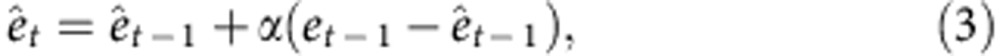


where *α* is a parameter that determines the degree of update based on the difference between the PE and the observed movement error. This update rule is rational when movement error shows trial-to-trial variability, as previously reported in an experimental study^27^, and movement error is observed with a sensory noise (detailed descriptions are given in the Update rule of PE section in Methods).

We also assume that the primitives encode the PE following a Gaussian: 
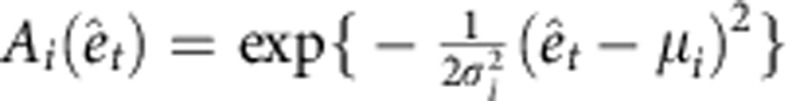
 (*ê*_*t*_ is the PE), where the scaling parameter *σ*_*i*_=*σ* is independent of *i* and *μ*_*i*_ε(−180°, 180°) is randomly sampled from a uniform distribution. The *i*th primitive is maximally activated when the PE is equivalent to its preferred PE *μ*_*i*_. A summarized procedure for the computer simulation is provided in the Summary of computer simulations section in Methods.

### Numerical simulation in randomly changing environments

**Figure 2:**
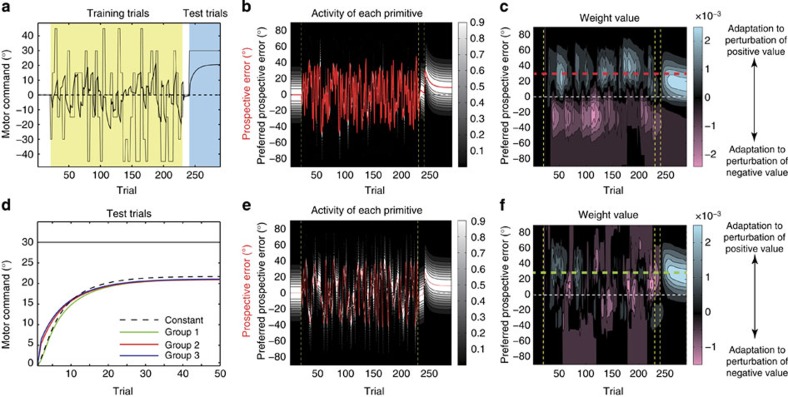
Structural learning. We investigated whether our model could explain the entire structural learning process. Each *p*_*t*_ was randomly sampled from the subset ***s***=(−45°, −30°, −15°, 0°, 15°, 30°, 45°) in the training trials. In groups 1, 2 and 3, the perturbation sequence varied in every trial, every two trials and every three trials, respectively. Washout trials were inserted between the training and test trials. These washout trials excluded the possibility that the movement error in the last training trial affects the learning speed in the test trials. During the test phase, a constant visuomotor rotation, ***p***=(30°, ⋯, 30°), was imposed. (**a**) Trial-by-trial change of *x*_*t*_ (thick line) and *p*_*t*_ (thin line) in group 2. (**b**) Activity of each primitive in group 2, where a strong white colour indicates high activity. The vertical axis shows the sorted preferred prospective error, *μ*_*i*_, from −90° to 90°. The red line denotes the prospective error. (**c**) Weighting parameters of each primitive in group 2. Blue and red colours indicate weighting parameters to compensate for perturbations of positive and negative values, respectively. The red dotted line shows that motor primitives for a 30° PE learn the 30° perturbation in the training trials. (**d**) Comparison of *x*_*t*_ in the adaptation to the visuomotor rotation among the three groups. Each *x*_*t*_ value is calculated by averaging across 100 simulations. (**e**) Activities of each primitive in group 1. (**f**) Weighting parameters of each primitive in group 1. The green dotted line shows that motor primitives for a 30° PE do not learn the 30° perturbation in the training trials.

Here, we try to observe the behaviour of motor learning under a stochastically changing environment. Our model predicts that learning speed in the test phase can be increased when the perturbation randomly varies in every two or three trials during the training trials (groups 2 and 3) (Fig. 2a,d). In contrast, learning speed was not facilitated when the perturbation randomly varied in every trial during the training trials (group 1). In group 2 (or 3), two (or three) consecutive identical perturbations make it more reliable to predict the movement error, and the primitives encoding the PE gradually acquire the knowledge to compensate for the same movement error (for example, primitives for 30° PE learn the 30° perturbation) (Fig. 2b and red dotted line in Fig. 2c). In the test phase, the motor memory embedded in the primitives for the positive PE is reactivated, which leads to an increase in learning speed. In contrast, when the perturbation changes from trial to trial (group 1), the PE does not have information regarding the perturbation because it was completely unpredictable (Fig. 2e and green dotted line in Fig. 2f), resulting in the failure of motor memory formation.

### Behavioural experiment

**Figure 3:**
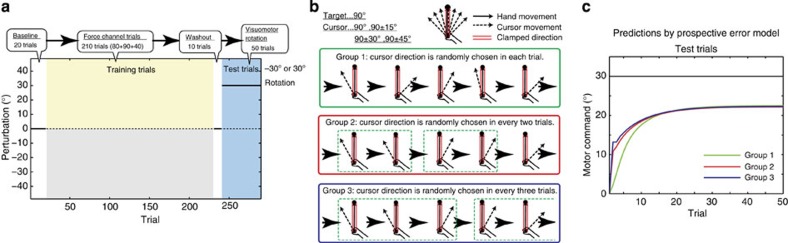
Schematic diagram of the behavioural experiment. (**a**) Subjects needed to adapt to a −30° or 30° visuomotor rotation after experiencing the force channel trials (see below). (**b**) Throughout the experiment, the target direction was fixed to 90°. In the force channel trials, the actual hand-movement direction was also fixed to 90° using a virtual wall (force channel trial). In groups 1, 2 and 3, the cursor movement varied randomly in every trial, every two trials and every three trials, respectively. (**c**) Prediction of our model in test trials (each *x*_*t*_ value is calculated by averaging across 100 simulations). In this simulation, *x*_*t*_ in each force channel trial is forcibly set to 0. The PE can be predicted more reliably in groups 2 and 3 than in group 1, and the motor learning is predicted to be facilitated in groups 2 and 3 compared with group 1: learning speed is significantly higher in groups 2 and 3 than in group 1.

It should be noted that the difference among groups 1, 2 and 3 described above is a novel prediction that has never been predicted nor tested. Therefore, we performed a behavioural experiment to validate this prediction. Notably, this prediction contrasts with a conventional Bayesian framework because, according to this framework, a more uncertain random perturbation is associated with faster learning in a subsequent adaptation to a constant perturbation^3^. In the present experiment, subjects moved a manipulandum to control a cursor on a horizontal screen towards a forward target. In training trials, the cursor’s movement direction randomly rotated either in every trial (group 1), in every two trials (group 2) or in every three trials (group 3) by a certain amount sampled from a set of rotations (−45°, −30°, −15°, 0°, 15°, 30° and 45°) (Fig. 3a,b). Hand movements during the training trials were always constrained along a straight line from the starting position to the target by the manipulandum (that is, force channel trial) (Fig. 3b), which allowed us to differentiate the predictions of our model from those of conventional models, as described below. After the training phase, subjects experienced a constant amount of visuomotor rotation (±30°) in test trials without the force channel. The training and test trials were interleaved with washout trials to rule out the possible effect of cursor movements in the last training trial on the learning speed in the test trials. Although this experimental setting was slightly different from the conditions we simulated in Fig. 2, the predictions of our model were invariant: learning speed in test trials was predicted to be faster in groups 2 and 3 than in group 1 (Fig. 3c; in these simulations, *x*_*t*_ in training trials was always set to 0 with assuming force channel trials).

**Figure 4:**
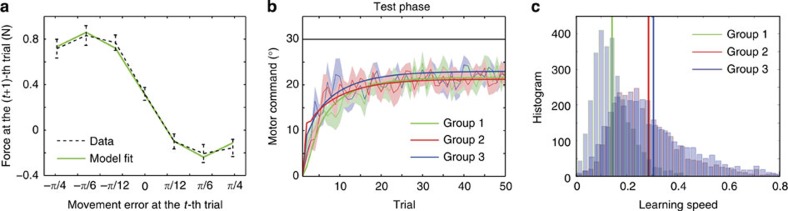
Results of our behavioural experiment. (**a**) Generated force at the (*t*+1)-th trial after experiencing a movement error *e*_*t*_ in the force channel trials in group 1 (black dotted line, mean±s.e.m., *n*=12). The green solid line shows the fitting of our model (*R*^2^=0.9950). (**b**) Actual data (mean±s.e.m., *n*=12 for each group) and learning curves predicted by our model (*R*^2^=0.8638 for group 1 (green), *R*^2^=0.7967 for group 2 (red) and *R*^2^=0.7968 for group 3 (blue)). Notably, the parameters were fit to data from only group 1, and our model predicted the learning curves for groups 2 and 3 with these parameters. Data for the adaptation to the 30° and −30° visuomotor rotations are included in each group. (**c**) Histogram of bootstrapped learning speed. Vertical solid lines denote the mean values of each distribution.

We used the force channel trials as training trials because they were useful to clarify the differences between our model and other conventional models. Although the force channel trials seem unnatural for an experimental setting, subjects can generate forces to compensate for the observed movement error (Fig. 4a). Because the force channel trials made identical target and hand-movement directions throughout all of the training trials, the same primitives were always activated according to the ideas from conventional models^10^,^11^,^12^,^13^. Because the average value of the movement error experienced by these primitives across many trials would be 0, the conventional models predict that no adaptation should occur. As several recent studies have suggested, motor adaptation could be influenced by reward^28^,^29^,^30^. In our experiment, however, the reward was likely to be almost identical among groups 1, 2 and 3 (the success rate was 1/7 in all the groups), suggesting no reward-associated difference in motor adaptation among the three groups. In contrast, because the PE was easily predicted in groups 2 and 3 compared with group 1, our model predicted that subjects in groups 2 and 3 would show faster adaptation during the test phase than those in group 1 (Fig. 3c).

The experimental results supported this prediction: in test trials, subjects in groups 2 (12 subjects: 6 for +30° rotation, 6 for −30° rotation) and 3 (12 subjects: 6 for +30° rotation, 6 for −30° rotation) demonstrated faster adaptation than those in group 1 (12 subjects: 6 for +30° rotation, 6 for −30° rotation), and subjects in group 3 demonstrated faster adaptation than those in group 2 (Fig. 4b). We fit an exponential function *e*_*t*_=*a* exp(−*bt*)+*c* to the bootstrapped data and estimated the learning speed *b*. The mean value of learning speed *b* was 0.1410 for group 1, 0.2845 for group 2 and 0.3037 for group 3 (Fig. 4c). Because these differences were significant (*P*<0.0001, randomization test), subjects in groups 2 and 3 were considered to adapt to visuomotor rotation faster than those in group 1, which was consistent with our model’s prediction.

Furthermore, we fit our model to the data from group 1 and tried to predict the data from groups 2 and 3 (details are provided in the Fitting our model to data from our experiment section in Methods). When we fit our model to the forces in force channel trials and the movement angles in test trials, *R*^2^ was 0.9950 and 0.8638, respectively (Fig. 4a,b). The movement angles in the test phase of groups 2 and 3 could be predicted with *R*^2^=0.7967 and *R*^2^=0.7968 (Fig. 4b).

**Figure 5:**
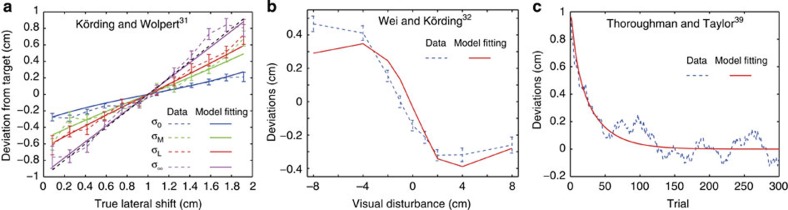
Model fitting to data in crcns.org. (**a**) Data from Körding and Wolpert^31^. Solid lines show the fit of our model (*R*^2^ was 0.9315, 0.9448, 0.9823 and 0.9786 for data of *σ*_0_, *σ*_*M*_, *σ*_*L*_ and *σ*_∞_, respectively). Dotted lines show actual data (mean±s.e.m., *n*=10). (**b**) Data from Wei and Körding^32^. Solid line shows the fit of our model (*R*^2^ was 0.8947). Dotted line shows actual data (mean±s.e.m., *n*=7). (**c**) Data from Thoroughman and Taylor^39^. Solid line shows the fit of our model (*R*^2^ was 0.8240). Dotted line shows moving average filtered actual data (mean, *n*=12).

In addition, when our model was used to fit the data sets from previous studies, the resulting *R*^2^ was higher than 0.8240 (Fig. 5, details are provided in the Fitting our model to data sets from previous studies section in Methods). These studies investigated phenomena seemingly unrelated to structural learning and our behavioural experiment, such as uncertainty effects^31^ or error size effects on error modification^32^, which were separately reproduced by different computational models, but our PE-based model could be fit to the data sets. Thus, we expect that the PE-based model will reproduce diverse features of motor learning in a unified manner.

### Reproduction of other phenomena

Here, we demonstrate that our PE-based model can also reproduce diverse phenomena that have previously been explained by different models. We used the best-fit parameters for group 1 in the numerical simulations described below.

### Effect of uncertainty on learning speed

Motor learning is hindered when the observed movement error includes uncertainty. For instance, motor-learning speed decreases when the end-point hand position is blurred^3^,^4^. In addition, increased blurring of the end-point position (higher uncertainty) is associated with slower learning speed. To explain this effect of uncertainty, previous studies used a Kalman filter^3^,^4^. Because the uncertainty in the observation of the movement decreases the Kalman gain and learning rate, the framework using a Kalman filter can explain how the uncertainty of the observation adversely influences the motor-learning speed.

**Figure 6:**
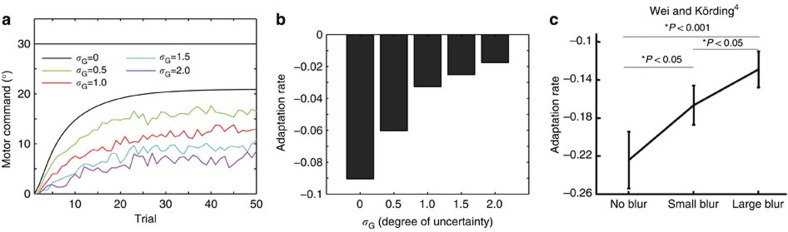
Uncertainty effect. To determine whether our model can explain an uncertainty effect, we simulated an experiment in which the model adapts to a 30° visual rotation for 50 trials with an observation noise, that is, *e*_*t*_=*p*_*t*_−*x*_*t*_+*ξ*_*t*_, where *ξ*_*t*_ is a Gaussian random noise with a mean of 0 and a variance of 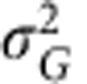. When *σ*_*G*_ is large, uncertainty is large for the observation of the movement error. (**a**) Trial-by-trial change of *x*_*t*_ averaged across 100 simulations. (**b**) Adaptation rate after fitting a state-space model *x*_*t*+1_=*Ax*_*t*_−*Be*_*t*_ to the simulated *x*_*t*_ shown in **a**, where *A* is a forgetting rate and *B* is an adaptation rate. (**c**) Previously reported adaptation rate (reproduced from Wei and Körding^4^).

Our model also reproduced the detrimental influence of the uncertainty of the error feedback on motor-learning performance (Fig. 6). The influence of the uncertainty can be interpreted based on a recursive equation of motor command (see the Recursive equation of motor command section in Methods for a detailed analysis):


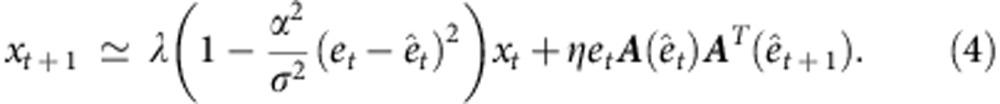


The learning rate is modulated by an inner product ***A***(*ê*_*t*_) ***A***^*T*^(*ê*_*t*+1_). The inner product is maximal when *ê*_*t*+1_=*ê*_*t*_ and minimal when *ê*_*t*+1_ is completely different from *ê*_*t*_; great inaccuracy of the prediction of the PE (that is, greater uncertainty of error feedback) is associated with reduced modulation of the learning rate.

### Savings

Savings is a phenomenon in which the adaptation to the second exposure is faster than that to the first exposure, although a washout is experienced after the first exposure^1^,^19^,^23^.

**Figure 7:**
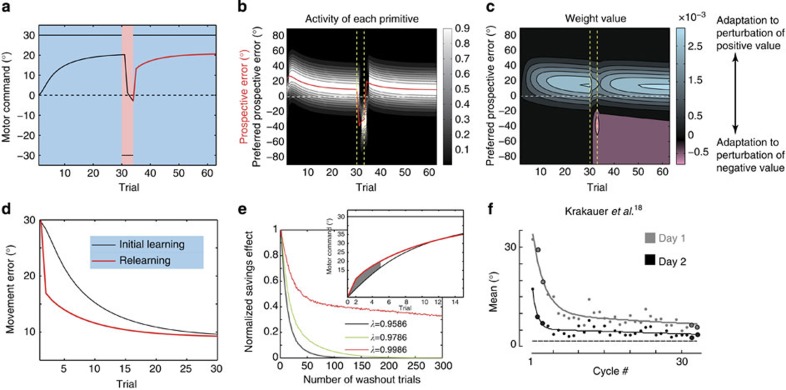
Savings. (**a**–**d**) To examine our model’s ability to explain the short-term savings effect, we considered an experiment in which a 30° visual rotation was applied for 30 trials (the initial learning phase), followed by a −30° visuomotor rotation for 5 trials (the opposite learning phase), and another set of the 30° visual rotation for 30 trials (the relearning phase). (**a**) Trial-by-trial change of *p*_*t*_ and *x*_*t*_ averaged across 10 simulations of short-term savings. (**b**) The activity of each primitive, with a strong white colour indicating high activity. The red line denotes the prospective error, *ê*_*t*_. Vertical dotted lines are drawn at the trials when the phases switched. The horizontal dotted line denotes the line on which *ê*_*t*_=0. (**c**) Weighting parameters of each primitive. Blue and red colours indicate weighting parameters to compensate for perturbations of positive and negative values, respectively. (**d**) Comparison of *x*_*t*_ between the initial learning and relearning phases. (**e**) Persistence of the savings effect and its dependence on the forgetting rate (*λ*=0.9586 (best-fit parameter for the data of group 1), 9786 or 9986). We simulated an experiment in which a 30° visual rotation was applied for 60 trials (the initial learning phase) followed by a 0° visuomotor rotation (washout phase), and another set of the 30° visual rotation was imposed for 20 trials (the relearning phase). The horizontal axis denotes the length of the washout trials. (Inset) comparison of *x*_*t*_ between the initial learning and relearning phases. We define the savings effect as the integral of the grey zone: the difference of *x*_*t*_ in the first five trials between the initial learning and relearning phases. This value should be 0 if there are no savings, and the value is positive when the learning speed in the relearning phase is higher than that in the initial learning phase. The savings effects were normalized by setting the maximal value to be 1. (**f**) Previously reported savings by Krakauer *et al.*^18^ (adapted by permission from Macmillan Publishers Ltd: Nature Neuroscience^18^, copyright 1999).

Figure 7a,d indicates the result of a simulation of an experiment in which subjects experience a 30°-visuomotor rotation (initial learning) followed by a −30°-visuomotor rotation (opposite learning) and then are exposed again to a 30°-visuomotor rotation (relearning). The −30°-exposure appears to eliminate motor memory, but the adaptation was faster in the relearning phase than in the initial learning, indicating that our model reproduced the savings. Notably, in contrast to previous models that adopt processes with multiple time constants (that is, slow and fast^1^,^2^,^20^), our model did not explicitly consider the presence of slow and fast states.

In our model, at the beginning of the initial learning phase, the motor primitives with preferred PEs close to 30° are activated (Fig. 7b) and the weighting parameters of these primitives are modified to decrease the movement error of the 30° rotation (Fig. 7c). However, as the adaptation proceeds, the movement error and the PE decrease, and as a result, different primitives are gradually involved in the decrement of the movement error (Fig. 7b). Because the motor primitives activated at the beginning of the initial learning phase are no longer activated during the latter half of the initial learning phase nor in the opposite learning phase, the weighting parameters of those primitives remain unchanged. Thus, when a 30°-perturbation was re-imposed in the relearning phase, the primitives maintaining the memory are reactivated, which contributes to accelerating adaptation to the 30°-perturbation relative to the initial learning phase.

Previous studies^19^,^20^ have also noted that even the two-state model comprising fast and slow processes, which was developed to explain the savings, cannot explain the experimental result that savings still exist even after a sufficient number of washout trials following the initial learning phase. As shown in Fig. 7e, even with a sufficiently long washout phase, our model can still account for the savings effect when the forgetting rate is close to 1.

### Anterograde interference

Anterograde interference is a phenomenon in which the adaptation to a novel environment (for example, clockwise visuomotor rotation) interferes with the subsequent adaptation to another novel environment (for example, counter-clockwise visuomotor rotation)^22^,^23^.

**Figure 8:**
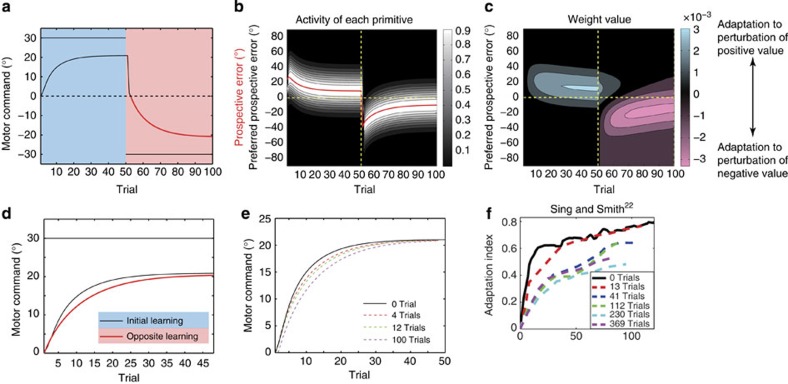
Anterograde interference. To determine whether our model could explain this effect, we simulated an experiment in which a 30° visual rotation for 50 trials (the initial learning phase) was followed by a −30° visuomotor rotation for 50 trials (the opposite learning phase). (**a**) Trial-by-trial change of *p*_*t*_ and *x*_*t*_ averaged across 10 simulations. (**b**) Activities of each primitive, with a strong white colour indicating high activity. The red line denotes the prospective error, *ê*_*t*_. Vertical dotted line is drawn at the trial at which the initial learning phase switches to the opposite learning phase. The horizontal dotted line denotes the line on which *ê*_*t*_=0. (**c**) Weighting parameters of each primitive. Blue and red colours indicate weighting parameters to compensate for perturbations of positive and negative values, respectively. (**d**) Comparison of *x*_*t*_ between the initial learning and opposite learning phases. In the opposite learning phase, the negative part of *x*_*t*_ is drawn (red line in **a**). (**e**) Trial-by-trial change of *x*_*t*_ in the opposite learning phase. Each dotted line denotes the dependence of *x*_*t*_ on the length of the initial learning phase. (**f**) Previously reported savings by Sing and Smiath^22^ (reproduced from a previous study^22^).

Figures 8a, d demonstrate the results of a simulation in which the subjects experienced a 30°-visuomotor rotation (initial learning) followed by a −30°-visuomotor rotation (opposite learning). Adaptation was slower in the opposite learning phase than in the initial learning phase, indicating that our model reproduced anterograde interference. The motor primitives whose preferred PEs were close to 0° were activated in the latter part of the initial and opposite learning phases (Fig. 8b). The weighting parameters of these primitives were modified to reduce the positive movement error in the initial learning phase, but the content of the motor memory of these primitives needed to be reversed for the opposite learning phase (Fig. 8c). This reversal may increase the number of trials needed for the adaptation in the opposite learning phase. In fact, a longer initial learning phase was associated with slower adaptation in the opposite learning phase (Fig. 8e).

### Spontaneous recovery

Motor memory is not easily eliminated once it is formed. After a sufficient amount of force-field training, a short exposure to the opposing force field appears to reverse the motor output (that is, the motor memory content). However, during the forgetting process of the motor memory, the motor memory for the originally trained force field can be spontaneously recovered^1^. This phenomenon is called spontaneous recovery^1^,^23^,^24^.

**Figure 9:**
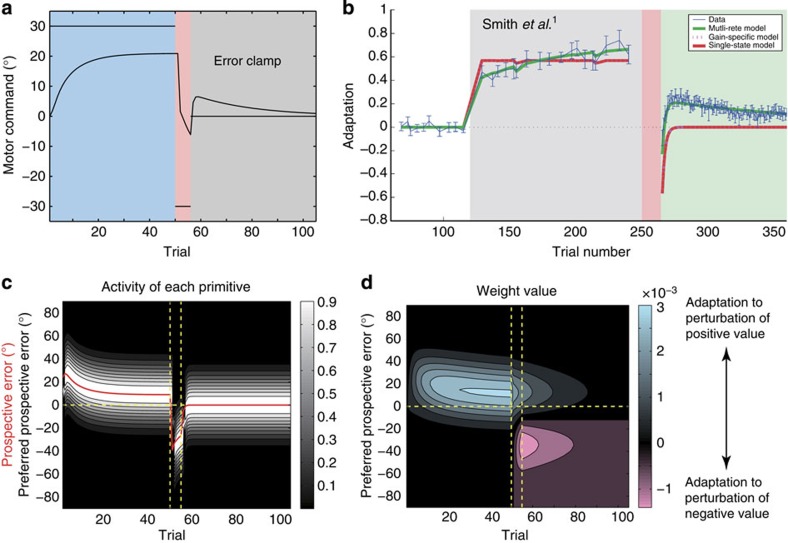
Spontaneous recovery. We simulated an experiment in which a 30° visual rotation for 50 trials (the initial learning phase) was followed by a −30° visuomotor rotation for 5 trials (the opposite force-learning phase), and error-clamp trials were imposed. In the simulation of the error-clamp trials, the movement error, *e*_*t*_, was forcibly set to 0°. (**a**) Trial-by-trial change of *p*_*t*_ and *x*_*t*_ averaged across 10 simulations. (**b**) Previously reported spontaneous recovery (reproduced from Smith *et al*.^1^). (**c**) Activities of each primitive, with a strong white colour indicating high activity. Vertical dotted lines are drawn for the trials when the phases switched. Horizontal dotted line denotes the line on which *ê*_*t*_=0. (**d**) Weighting parameters for each primitive. Blue and red colours indicate weighting parameters to compensate for perturbations of positive and negative values, respectively.

Figure 9a indicates the result of a simulation in which the subjects experienced a 30°-visuomotor rotation (initial learning phase) followed by a brief period of a −30°-visuomotor rotation (opposite learning phase) and finally a series of error-clamp trials in which the movement error was constrained to 0 (error-clamp trials). At the end of the opposite learning phase, the motor memory for the 30°-visuomotor rotation appeared to be completely eliminated, but the motor memory re-emerged during the error-clamp trials, indicating that our model successfully reproduced spontaneous recovery.

A sufficient amount of initial training trials resulted in a PE of almost 0, and almost all of the motor primitives involved in compensating for the 30°-visuomotor rotation had preferred PEs that were close to 0 (Fig. 9c). However, during the subsequent opposite learning phase, the number of training trials was small and the adaptation was accomplished while the PE did not converge to 0. Thus, the motor primitives involved in the opposite learning phase had PEs that were different from 0, indicating that the motor memory formed in the initial learning phase was not overwritten (Fig. 9d). In the error-clamp trials, the PE gradually approached 0, which reactivated the motor memory embedded in the motor primitives involved in the initial learning phase, leading to a spontaneous recovery of the motor memory.

## Discussion

We propose a novel motor-learning model based on motor primitives. Our model assumes that each primitive is activated by a PE, based on both theoretical consideration of how motor memory can be formed and maintained in a randomly varying environment and previous neurophysiological findings showing that some neurons encode a PE for online movement control^16^,^17^. To validate our model, we confirmed its novel prediction that motor-learning speed in response to a constant amount of perturbation is increased after experiencing the same movement errors in two or three consecutive trials. This phenomenon cannot be predicted by conventional computational models, assuming that the recruitment of the motor primitives is determined only by the planned movement direction^10^,^11^,^12^, by Bayesian framework^3^ nor by reinforcement learning based on ‘reward’^28^,^29^,^30^. In addition, this facilitatory effect cannot be explained by a previous model where an update of the motor command depended on the executed movement directions^13^, because the hand-movement direction in our experiment was kept identical to the target direction using the force channel. Although it is possibile that the update of the motor command depends on the cursor movement directions (see Discussion in Gonzalez-Castro *et al.*^13^), this framework cannot solely explain why a blurred end-point position decreases the learning rate; if movement error is linearly processed, the ensemble-averaged movement errors are the same between blurred and non-blurred conditions, *E*[*e*_*t*_+*ξ*_*t*_]=*E*[*e*_*t*_], where *ξ*_*t*_ denotes uncertainty. In contrast, our behavioural experiment validated our novel prediction (Fig. 4).

Our model also has strong power to explain a wide variety of other motor-learning-related phenomena^1^,^2^,^3^,^4^,^5^,^6^,^7^,^8^,^19^,^20^,^22^,^23^. Although different models have been conventionally proposed to explain different types of phenomena, our model can explain these phenomena in a unified manner (that is, in a single model with the same parameters) (Figs 2 and 6, 7, 8, 9).

To account for phenomena such as savings, anterograde interference and spontaneous recovery, recent computational studies have proposed that a motor memory has multiple time constants (that is, fast and slow processes^1^,^2^,^20^,^22^,^33^). Conversely, our model does not explicitly assume the presence of fast and slow motor-learning processes. Nevertheless, our model was able to account for these motor-learning phenomena, in addition to other types of phenomena that multiple timescale models cannot explain, such as structural learning or the change in learning rates due to uncertainty.

The explanatory power of our model is derived from the determination of the recruitment pattern of motor primitives based on the trial-by-trial variation of the PE. When the movement error is positive in consecutive trials, the PE is also predicted to be positive, and this positive PE activates a group of motor primitives responsible for compensating for the positive movement error. In these trials, a group of motor primitives responsible for compensating for a negative movement error remains inactivated and maintains the motor memory compensating for a negative movement error (Figs 7a and 9a). In contrast, a group of motor primitives for a near-zero PE is activated in the latter part of the learning phase independent of whether the movement error is positive or negative (Fig. 8a). Therefore, the motor primitives for a large PE are recruited in a task-dependent manner, but only at the beginning of the learning phase, whereas those for a small PE are recruited in a task-independent manner, but only in the latter part of the learning phase. The PE-dependent recruitment pattern of motor primitives explains why our model can reproduce savings, anterograde interference and spontaneous recovery. Furthermore, simulated relearning curves in Fig. 7d can be observed in an experiment in which subjects can use cognitive strategy to correct errors^34^. Our model indicates that cognitive strategy can be partly explained from a mechanistic viewpoint.

Similarly, this recruitment feature can also explain why the trial-dependent characteristics of the perturbation influence the learning rate. When the perturbation changes from trial to trial, the PE also randomly fluctuates, activating different sets of motor primitives, which lead to a lower learning rate because the formation of the motor memory is distributed across a large portion of motor primitives. Conversely, when the perturbations are more predictable, such as when identical perturbations are repeated in consecutive trials, the PE can be more reliably predicted. This predictability of the PE activates the same sets of motor primitives, and thus the formation of the motor memory is concentrated in a small portion of motor primitives, leading to a higher learning rate. These results suggest a novel interpretation for how the brain processes movement-error information; the movement error is used both for motor learning and for determining which primitives are recruited for that motor learning.

It is well known that when a visuomotor rotation is abruptly imposed, the amount of motor-command correction in the subsequent trial is not proportional to the amount of rotation; rather, it decreases with the amount of rotation^32^. This phenomenon was previously explained by a Bayesian framework^32^ in which a larger the visuomotor rotation was associated with a larger difference between the planned cursor movement direction and the executed hand-movement direction, resulting in a decreased learning rate. However, when the amount of visuomotor rotation is gradually increased, such a reduction in the learning rate is not observed^35^. The different adaptation behaviours between abrupt and gradual applications of visuomotor rotation can also be explained by our model framework. In the case of gradual visuomotor rotation, the movement error is very small and the PE is reliably predictable. Thus, the same group of motor primitives is always recruited, indicating that the learning rate is not affected by the difference between planned and executed movement directions. By contrast, abrupt visuomotor rotation results in greater movement error and the PE changes considerably, leading to a decrease in the learning rate.

**Figure 10:**
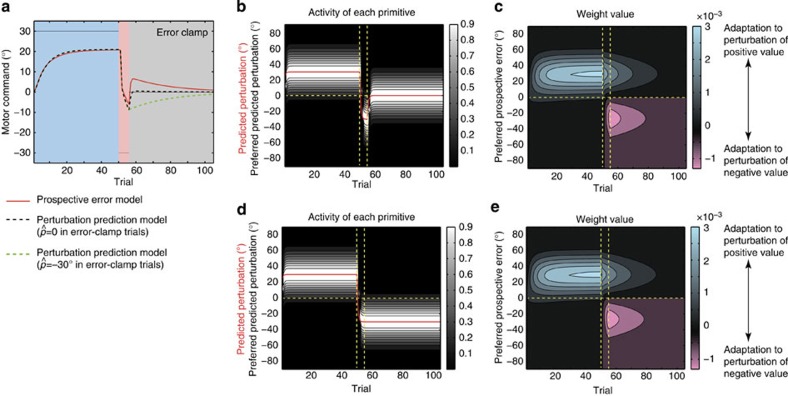
Comparison of the prospective error model and perturbation prediction model. (**a**) Trial-by-trial change of *p*_*t*_ and *x*_*t*_ averaged across 10 simulations. The grey zone denotes error-clamp trials in which the error, *e*_*t*_, was forcibly set to 0°. (**b**) Activities of each primitive in the perturbation prediction model when 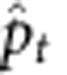 is forcibly set to 0° in error-clamp trials, with a strong white colour indicating high activity. Red line denotes predicted perturbation. Vertical dotted lines are drawn for the trials when the phases switched. The horizontal dotted line denotes the line on which 
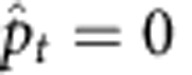
. (**c**) Weighting parameters of each primitive when 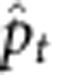 is forcibly set to 0° in error-clamp trials. Blue and red colours indicate weighting parameters to compensate for perturbations of positive and negative values, respectively. (**d**) Activities of each primitive in the perturbation prediction model when 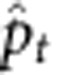 is forcibly set to −30° in error-clamp trials. (**e**) Weighting parameters of each primitive when 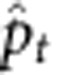 is set to −30° in error-clamp trials.

We have theoretically shown that motor primitives should encode the information of *p*_*t*_. In our model framework, however, we assumed that the motor primitives encode the prediction of *e*_*t*_ rather than the prediction of *p*_*t*_ itself, because *e*_*t*_ contains some information regarding *p*_*t*_. Interestingly, a model in which the PE determines ***A***_*t*_ has stronger explanatory power than a model in which the predicted *p*_*t*_ determines ***A***_*t*_ (Fig. 10).

We also assumed that the PE is updated based on a simple linear updating equation with a constant *α* (equation (3)), but other candidates can be considered. An example is the Kalman filter^36^, in which *α* can be modulated in each trial by uncertainty. In addition, *ê*_*t*_ can be updated based not only on *ê*_*t*−1_, but also *ê*_*t*−2_, *ê*_*t*−3_ or a longer history of *ê*. Although a simple linear update of the PE is sufficient to reproduce many simulated phenomena in this study, we expect that the Kalman filter and a longer history will have stronger explanatory power than equation (3). Further study is needed to investigate how the PE is updated.

Our model was confirmed by an experiment involving only a 10-cm (ballistic) reaching movement. Thus, the current aspects may or may not be applicable to more general movements such as longer reaching movements and three-dimensional reaching. Future studies will be necessary to answer this problem, but we believe that the present ideas are also applicable to those movements, considering that the aspects of motor learning revealed by previous studies using the same experimental set-up have been confirmed for the other movements such as saccadic adaptation^37^ and locomotion^38^.

Furthermore, for simplicity, this study addressed with reaching movements towards a single target. However, we need to expand our model into one that can account for movement towards multiple targets. Adaptation effects in a reaching movement towards a single training target are generalized to movements towards other spatially distributed targets^10^,^11^. The degree of generalization depends on the angular difference between the trained and tested target directions. To explain this generalization effect, one possible idea is to extend from a univariate function *A*_*i*_(*ê*_*t*_) to a bivariate function *A*_*i*_(*d*_*t*_, *ê*_*t*_), where *d*_*t*_ is a target direction. There are several candidates for these extensions. For example, the PE and desired movement direction could be either additively integrated, that is, *A*_*i*_(*d*_*t*_, *ê*_*t*_)=*f*_*i*_(*d*_*t*_)+*g*_*i*_(*ê*_*t*_) (*f*(·) and *g*(·) are functions), or multiplicatively integrated, that is, *A*_*i*,*t*_(*d*_*t*_, *ê*_*t*_)=*f*_*i*_(*d*_*t*_)*g*_*i*_(*ê*_*t*_). Although recent studies support the multiplicative interaction as a strong candidate for the integration of multiple variables^14^,^15^, this idea needs to be validated by conducting additional experiments.

## Methods

### Theoretical analysis

The averaged update rule across all possible realizations can be written as


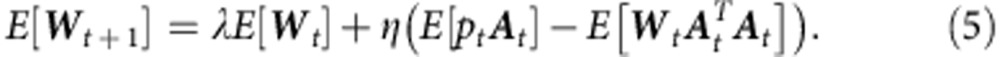


After many trials, *E*[***W***_*t*+1_] and *E*[***W***_*t*_] converge to ***W***, and we obtain equation (2). If *p*_*t*_ is independent of ***A***_*t*_ and *E*[*p*_*t*_]=0, *E*[*p*_*t*_***A***_*t*_]=*E*[*p*_*t*_]*E*[***A***_*t*_]=0 and 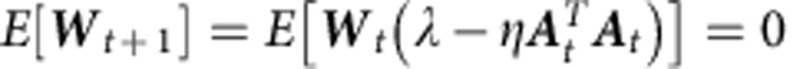 because *E*[***W***_0_] is 0. Thus, motor primitives can form and maintain motor memory in a randomly varying environment when ***A***_*t*_ is correlated to *p*_*t*_.

### Update rule of PE

Prospective error is a predicted movement error based on the current prediction and the prediction error between the current prediction and the observed movement error. When the observed movement error is *e*_*t*_ and the true (noiseless) movement error is *g*_*t*_, the observation process can be written as *e*_*t*_=*g*_*t*_+*ξ*_*t*_, where *ξ*_*t*_ is the observation noise (sensory noise). Here, we assume a Gaussian noise whose mean is 0 and variance is 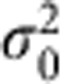 as the observation noise. Recent studies reported that, even when there is no perturbation, movement error shows trial-to-trial variability^27^. If the variability of movement error is available in our motor system (that is, our motor system can utilize a generative model of movement error *g*_*t*+1_=*g*_*t*_+*ζ*_*t*_ (*ζ*_*t*_ is a Gaussian noise whose mean is 0 and variance is 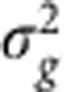)), our motor system can optimally predict the movement error in the next trial following


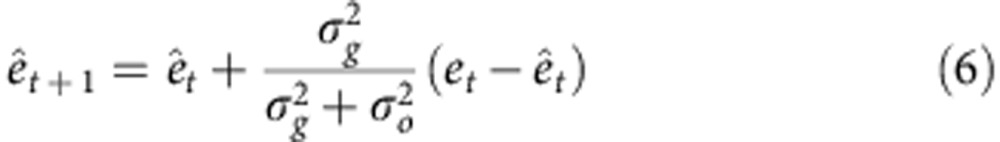


to minimize the variance of prediction error. Equation (3) is thus an optimal update of the PE when 
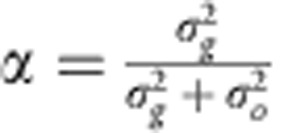
. Notably, this update rule is equivalent to a Kalman filter^36^, but we did not assume any update of 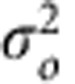 and 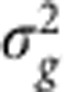 for simplicity (see Discussion).

### Recursive equation of motor command

We can derive the recursive equation of motor command (state-space representation of motor learning) when movement error decreases gradually. In this case, ***A***(*ê*_*t*+1_)=***A***(*ê*_*t*_+*α*(*e*_*t*_−*ê*_*t*_))≃***A***(*ê*_*t*_)+*α**A***′(*ê*_*t*_)(*e*_*t*_−*ê*_*t*_), where ***A***′ is the derivative of ***A***. When *A*_*i*_ is a Gaussian, multiplying the update equation of ***W***_*t*_ (equation (1)) by ***A***^*T*^(*ê*_*t*+1_) yields


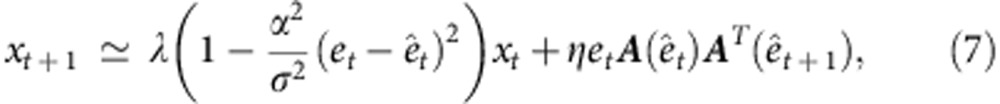


where the learning rate is modulated by the inner product ***A***(*ê*_*t*_)***A***^*T*^(*ê*_*t*+1_). The inner product can be further calculated as 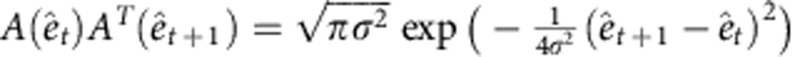, where *N*→∞ and *μ*ε(−∞,∞) are assumed. The recursive equation can be rewritten as:


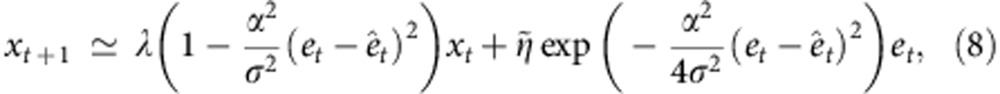


where 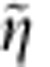 is 
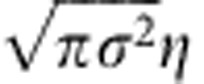
 and both the forgetting and learning rate are modulated by (*e*_*t*_−*ê*_*t*_)^2^. Therefore, a more predictable PE is associated with higher forgetting and learning rates (slower forgetting and faster learning).

### Summary of computer simulations

By setting *ê*_0_=*e*_0_=0 and ***W***_0_=0, our simulation consisted of the following four steps:


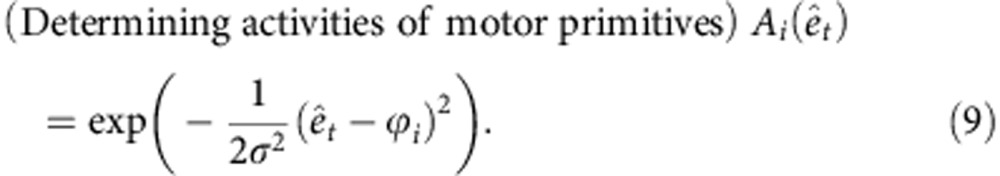



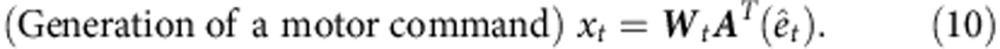



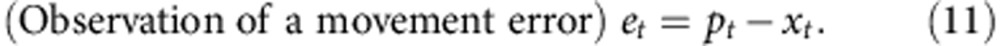



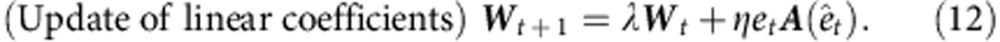



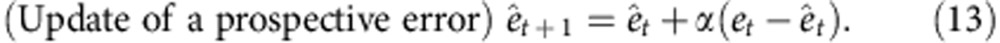


### Fitting our model to data from our experiment

Our model has four parameters: a forgetting rate *λ*, a learning rate *η*, an update rate of PE *α* and a width of motor primitives *σ*. First, assuming ***W***_*t*_=0 and *ê*_*t*_=0, we determined *α* and *σ* by fitting the amount of error modification 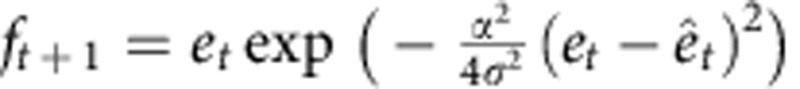 (equation (8)) to the data in training trials of group 1 (Fig. 4a, *R*^2^=0.9950), because *f*_*t*+1_ is uncorrelated to *e*_*t*_ only in group 1. The assumptions, ***W***_*t*_=0 and *ê*_*t*_=0, can be assumed only in data from group 1, because the average error in training trials of group 1 is 0 as a result of completely random cursor movements. Because the data were related to generated force and our model focused on movement direction, we scaled the equation, *mf*_*t*+1_+*n* to fit for the data (*m* and *n* were best-fit parameters). This fitting yielded the best-fit *σ*/*α*=0.3586 × (360/2*π*), that is, we could not separate *α* and *σ* based on this data fitting. Next, we searched the best-fit *λ*, *η*, *α* and *σ* for the learning curve for group 1 in test trials, resulting in *λ*=0.9586, *η*=2.3913, *α*=0.8 (we searched the best *α* by setting *α*=0, 0.1, 0.2, ..., 0.9, or 1.0) and *σ*=0.2868 × (360/2*π*). Notably, we fit all of the parameters to the data from group 1 (*R*^2^=0.8638). However, our model can also predict the data from groups 2 and 3 (*R*^2^=0.7967 and *R*^2^=0.7968).

### Fitting our model to data sets from previous studies

We fit our model to conventional data in ( http://crcns.org): data from Körding and Wolpert^31^, Wei and Körding^32^ and Thoroughman and Taylor^39^. Parameters *σ* and *α* were set to the best-fit parameters for our experimental data, *σ*/*α*=0.3586 × (360/2*π*) and *α*=0.8. The best-fit forgetting and learning rates *λ* and *η* were identified for each data set.

### Data from Körding and Wolpert

When error feedback includes uncertainty, the learning rate in our model is modulated by 
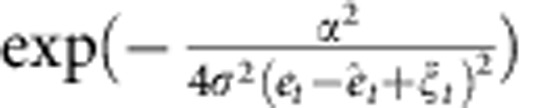
 (equation (8)). If this factor is averaged across all of the possible uncertainty values, *ξ*_*t*_, simple calculations yield 
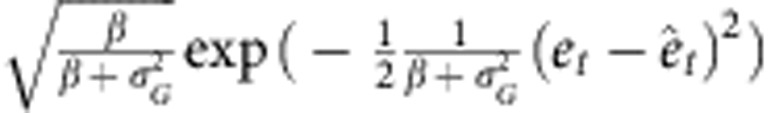
; therefore, the amount of error modification is 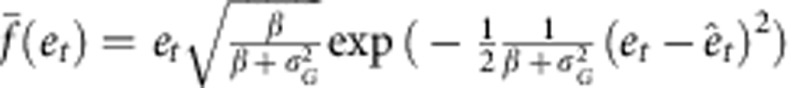. We scaled this equation, 
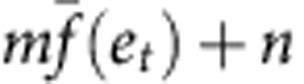
, to fit the data of Körding and Wolpert^31^, assuming that *ê*_*t*_=0 (this assumption is correct because the averaged error across all of the trials was almost zero), *σ*_*G*_=(18°, 30°, 36° and 60°) in the *σ*_0_, *σ*_M_, *σ*_L_ and *σ*_∞_ conditions, respectively (Fig. 5a). Because our model focused on movement direction and their data focused on movement deviation, this scaling was necessary. *R*^2^ was 0.9315, 0.9448, 0.9823 and 0.9786 for data of *σ*_0_, *σ*_*M*_, *σ*_*L*_ and *σ*_∞_, respectively.

### Data from Wei and Körding

We calculated the relationship between motor command at the (*t*+1)-th trial, *x*(*t*+1) and perturbation at the *t*-th trial, *p*(*t*), when the perturbation in each trial was randomly sampled from *p*=(−45°, −30°, −15°, 0°, 15°, 30°, 45°). This simulation was conducted for 30 simulation runs and 210 trials in each simulation run (the weight parameter ***W*** was reset to 0 at the beginning of each simulation run). When we compared the scaled motor commands *mx*(*t*+1)+*n* to the data of Wei and Körding^32^ (Fig. 5b), *R*^2^ was 0.8947.

### Data from Thoroughman and Taylor

Data from Thoroughman and Taylor^39^ were related to adaptation to a curl force field with 16 targets. Because we did not consider multiple targets in our model (see Discussion), we fit our model to their data after moving average filtering. The size of the filter was 16 and weight was uniform, that is, the filtered error at the *t*-th trial *ē*_*t*_ was 
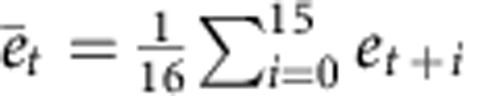
, where *e*_*t*_ represents movement error without the filtering. This filter can be expected to minimize the effect of the generalization of learning effects across different target directions. Figure 5c shows the filtered error. We scaled the movement error in our model, *me*(*t*)+*n*, to fit to their data. *R*^2^ was 0.8240.

### Perturbation prediction model

We theoretically proved that ***A***_*t*_ should encode the information for perturbation *p*_*t*_. Here, we assumed a perturbation prediction model in which ***A***_*t*_ is determined by 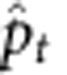, where 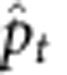 is a predicted perturbation and updated by 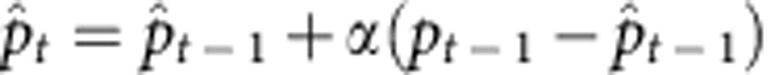. We compared the PE model and the perturbation prediction model based on numerical simulations of spontaneous recovery (Fig. 10). Because we are not sure how the subjects predicted *p*_*t*_ in error-clamp trials, 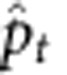 was forcibly set to 0 or −30 (perturbation just before the error-clamp trials).

### Behavioural experiment

Thirty-six healthy, right-handed volunteers (22 males, 14 females, aged 18–38 years) participated in this study and were paid for their time. The participants were pseudo-randomly assigned to one of the six experimental groups, group 1 CW, group 1 CCW, group 2 CW, group 2 CCW, group 3 CW or group 3 CCW, where CW indicates clockwise rotation (−30° rotation) and CCW indicates counter-clockwise rotation (30° rotation). The numbers of females and males were the same in group 1 CCW and in group 2 CCW (three males and three females) and among group 1 CW, group 2 CW, group 3 CW and group 3 CCW (four males and two females). The subjects had no cognitive or motor disorders and were naïve to the concept of visuomotor rotation and the purpose of the experiment. All participants were clearly informed of the experimental procedures in accordance with the Declaration of Helsinki and provided written informed consent before the experiment began. All procedures were approved by the ethics committee of the Graduate School of Education at the University of Tokyo.

Participants were asked to make pointing movements with their right arm while holding the handle of the manipulandum (Phantom 1.5 HF; Geomagic, Rock Hill, SC, USA). The handle position was displayed as a white cursor (a 6-mm circle) on a black background on a horizontal screen located above their hand. The movement of the handle was constrained to a virtual horizontal plane (10 cm below the screen) that was implemented by a simulated spring (1.0 kN m^−1^) and dumper (0.1 N per (m s^−1^)). A brace was used to reduce unwanted wrist movement. Upper trunk motion was constrained by a harness. Before each trial, participants were required to hold the cursor at its starting position (a 10-mm circle). After a 2-s holding time, a grey target (a 10-mm circle) appeared. After an additional randomly selected holding time (250–350 ms), the target colour changed to purple, signalling the participant to initiate a pointing movement. Subjects were required to move the handle with a peak velocity of 470±45 mm s^−1^ (the target velocity was calculated using the minimum-jerk theory with a movement amplitude of 10 cm and a duration of 0.4 s). A warning message appeared on the screen if the movement velocity of the handle rose above (‘fast’) or fell below (‘slow’) this threshold value. Subjects were also required to move the handle with an amplitude of 10 cm. When the movement amplitude was 10 cm, the sound of an explosion was produced. At the end of each trial, the handle was automatically moved back to the starting position by the manipulandum.

In training trials (force channel trials), we used the ‘error-clamp’ method^1^,^40^,^41^. During error-clamped trials, the trajectory of the handle was constrained to a straight line towards the target by a virtual ‘channel’ in which any motion perpendicular to the target direction was constrained by a one-dimensional spring (2.5 kN m^−1^) and damper (25 N/(m/s)).

Manipulandum motion data were recorded at a sampling rate of 500 Hz. Motion data were low-pass filtered using a fourth-order Butterworth filter with a 10-Hz cutoff. Movement onset time was defined as the first time point during which hand-movement velocity first exceeded 10% of its peak value for at least 50 ms.

For the second trial of the test trials with visuomotor rotation, one of the 12 subjects in group 2 showed an outlying behaviour. The mean movement angle in group 2 at the trial *μ* was 27.6944, the s.d. *σ* was 11.6704 and the movement angle of this subject in this trial was 62.8017, which is larger than *μ*+3*σ*. Thus, we eliminated this outlying data point from our analysis. Notably, this elimination of the outlier did not affect our results at all.

To determine whether learning speed was different among groups 1 (CCW and CW), 2 (CCW and CW) and 3 (CCW and CW), we conducted a bootstrap sampling and a randomization test. For bootstrap sampling, the learning speed was sampled 3,000 times in each group, and we calculated the mean value of the 3,000 sampled learning speeds. To determine whether the mean values of each group were significantly different, randomization tests were conducted. In each randomization test, the bootstrap-sampled learning speeds in groups 1 and 2 (1 and 3, or 2 and 3) were intermingled and randomly divided into two groups. We calculated the difference in the mean values of each randomized group and counted how many times this difference was larger than the difference of the mean learning speed (0.1410 for group 1, 0.2845 for group 2 and 0.3037 for group 3) to calculate *P*-values for the randomization tests.

## Author contributions

K.T. and M.H. designed and performed the experiments. K.T. performed the analyses and wrote the manuscript. D.N. oversaw the experiments, analyses and writing.

## Additional information

**How to cite this article:** Takiyama, K. *et al.* Prospective errors determine motor learning. *Nat. Commun.* 6:5925 doi: 10.1038/ncomms6925 (2015).
